# Real-Time Fault Classification for Plasma Processes

**DOI:** 10.3390/s110707037

**Published:** 2011-07-06

**Authors:** Ryan Yang, Rongshun Chen

**Affiliations:** Department of Power Mechanical Engineering, National Tsing Hua University, Hsinchu 30013, Taiwan; E-Mail: ryyanga@gmail.com

**Keywords:** process/equipment fault detection, fault classification, optic emission spectrum (OES)

## Abstract

Plasma process tools, which usually cost several millions of US dollars, are often used in the semiconductor fabrication etching process. If the plasma process is halted due to some process fault, the productivity will be reduced and the cost will increase. In order to maximize the product/wafer yield and tool productivity, a timely and effective fault process detection is required in a plasma reactor. The classification of fault events can help the users to quickly identify fault processes, and thus can save downtime of the plasma tool. In this work, optical emission spectroscopy (OES) is employed as the metrology sensor for *in-situ* process monitoring. Splitting into twelve different match rates by spectrum bands, the matching rate indicator in our previous work (Yang, R.; Chen, R.S. *Sensors* **2010**, *10*, 5703–5723) is used to detect the fault process. Based on the match data, a real-time classification of plasma faults is achieved by a novel method, developed in this study. Experiments were conducted to validate the novel fault classification. From the experimental results, we may conclude that the proposed method is feasible inasmuch that the overall accuracy rate of the classification for fault event shifts is 27 out of 28 or about 96.4% in success.

## 1. Introduction

Due to the rapid progress in the semiconductor fabrication process, the density of electronic devices in the same die area is increasing significantly. At the same time, the process flows for manufacturing a device become more complicated. To improve the device yields, the process windows must be greatly reduced, compared to the micro size device fabrication, and the fabrication equipment has to be designed to meet the narrow process window. As a result, the sophisticated semiconductor fabrication equipment, usually costing several millions of US dollars, has inherent variability in process condition control. If the plasma process is halted due to some process fault, the productivity will be reduced and the device cost will increase substantially. In order to maximize the process/wafer yields, the fault process, that is, any significant shift in the process conditions, must be detected when the variation becomes large, compared to the process background noise. When the operating conditions shift beyond an allowable range, which will decrease the product yield, timely and accurate fault detection is required in semiconductor manufacturing [1]. The classification of fault events can help the users to quickly identify the faulty process, and thus can minimize or avoid plasma tool downtime.

To identify and thus to classify the causes of faults is equally critical as sensing the plasma etching faults because a rapid classification and corrective action will minimize the tool downtime and increase the tool productivity. In semiconductor manufacturing, after a fault is detected, the cause is identified in two ways. The first way is to use a trial-and-error approach, inspecting all events associated with the fault. Therefore, the tool downtime and corrective cost are often dependent on the working experience of equipment engineers or technicians. The trial-and-error approach is slow and tedious, and is not a cost effective solution. Unfortunately, this corrective approach has been widely used for troubleshooting tool faults by the most semiconductor manufacturers. The second method involves the addition of some measurement tools and sensors. By analyzing the process data obtained by the sensors, one may find the probable causes of a fault. The second method is still slow and costly since the engineers may have to analyze a large amount of data before finding the possible solution. Furthermore, the success of finding the clues still relies heavily on the workers’ experience and understanding of the process.

Recently, fault classification with automatic pattern recognition has been proposed as a new method. Bunkofske *et al.* [2] used T^2^ contribution plots to represent a specific pattern or fingerprint, which can be used to classify and to diagnose the faults by identifying the measured parameters, the most likely cause of failure. Ison *et al.* [3] utilized probabilistic models, based on historical data, such as tree-based or generalized linear models, for automatically classifying faults. Love and Simaan [4] described the use of a knowledge-based approach for fault diagnosis. May and Spanos [5] proposed a methodology for automatically detecting the faults through knowledge-based and probabilistic modeling. Generally, the aforementioned methods identified the fault signature first, and then compared it to previous fault signatures that have already been classified to identify the possible cause [6–11]. The disadvantages of those methods are that they needed a much longer computing time and can hardly achieve a real-time classification. In this study, a real-time classification method for fault plasma process is developed to quickly and accurately identify the causes of process faults such that the process tool downtime can be reduced significantly to improve the wafer/product yield and cost.

## 2. Experimental Setup

### 2.1. Plasma Condition Monitor Sensor

**Figure 1.**
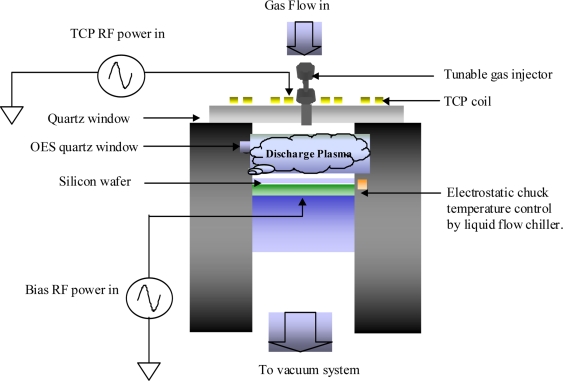
The configuration of the TCP.

As shown in Figure 1, a Transformer Coupled Plasma (TCP) reactor, which is a high-density-plasma source and is utilized to perform the etching processes, was used as the test vehicle in this work. The TCP uses RF power to activate its coil for generating an electromagnetic field, which can ionize the gases to be used in etching process. The bias RF power is also employed to control the ion bombardment force in the wafer chuck to obtain the required etching properties. A TCP reactor is typically operated below 100 mtorr with top and bottom 13.56 MHz RF generators in the TCP coil and electrostatic chuck, respectively. To sustain the uniformity of the photoresist pattern, the wafer temperature is controlled by a backside helium cooling system during the process stage. The helium gas, an intermediate material, is employed to transfer heat into the electrostatic chuck (ESC). The heat is removed by dielectric liquid, which flows into the ESC, and can keep the temperature at a certain level using a temperature control unit.

OES is an *in-situ* sensor for plasma process monitoring, which is designed as a non-invasive technique to avoid disturbing the plasma etching. The plasma emission lights have rich information about the plasma species, which can be used to monitor the etching rate, uniformity, selectivity, critical dimensions, and even the profile of etching features on a wafer. The OES monitoring wavelength covers roughly from 190 to 870 nm. The OES uses a 2,048-pixel CCD array with an optical resolution of about 1.3 nm for the monitoring system. The intensity of CCD reading is affected by the snapping time, set to be 100 ms in this study. The optic fiber, a bundle with seven 100-μm diameter fibers and terminated on each end by SMA 905 connectors, is utilized to assure that the light will be transmitted into the OES unit adequately, even if some of the fibers are broken due to excessive stress. Each optical adapter consists of a fiber-packed co-monitoring device with a UV collimating mirror and a connection port for the TCP reactor fiber assembly.

### 2.2. Data Collection

**Figure 2.**
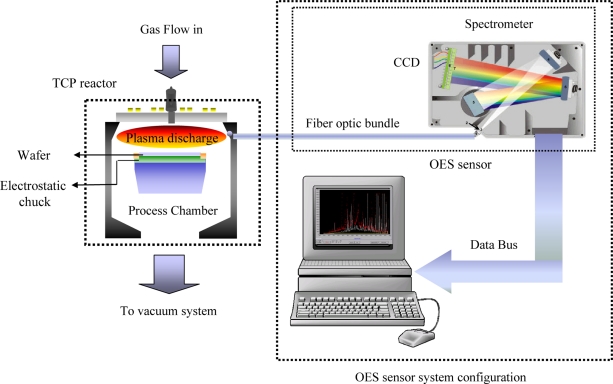
Overall configuration of the experimental system.

Figure 2 shows the overall configuration of the experimental system, which includes a TCP reactor, an OES module, and a computer. Through the optic fiber, the plasma emission beam is sent out from the OES window into the OES module. The plasma light incides onto the grating spectrum device, in which the split light is detected by the CCD unit of spectrometer, used to sense the wavelength intensity and transfers the corresponding data to the computer for further analysis. The spectrum data of the whole recipe are collected and then integrated time by time.

**Figure 3.**
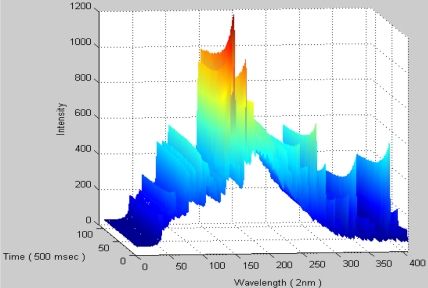
Spectrum data integrated with time.

Figure 3 shows an example of the spectrum data integrated by time, representing the full process response for both transient and steady states. In the transient state, the molecular/atom of the plasma is excited and ionized by RF power, which may induce plasma spiking, RF reflecting power, unstable pressure, and so on. In nano-scale fabrication processes, this transient state should be of concern due to the narrow critical process window. However, the amount of process data is huge and cannot be easily handled by a simple computation method. For effectively controlling the data, the sampling time is set to be 500 ms in the experiments.

### 2.3. Method of Fault Type Classification

A real-time plasma process fault detection method has been developed in our previous study [12]. It employed a plasma condition matrix, which was compared with the healthy plasma model by using the time series of the OES full spectrum intensity data. An indicator, which is called sigma model matching rate, was employed to show the differences between the normal and abnormal plasma conditions. It only can detect the abnormal plasma process; however, a real-time fault classification function is needed to find the cause of faults to reduce the process tool troubleshooting time.

**Figure 4.**
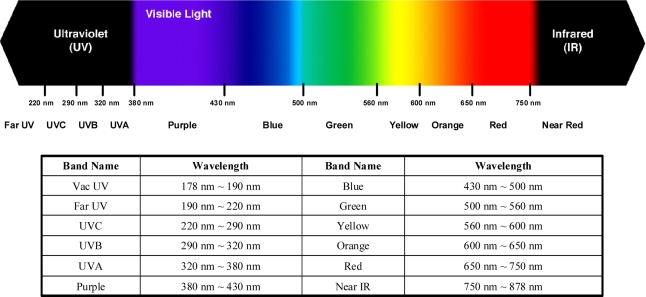
Definition of spectral bands from OES sensor.

**Figure 5.**
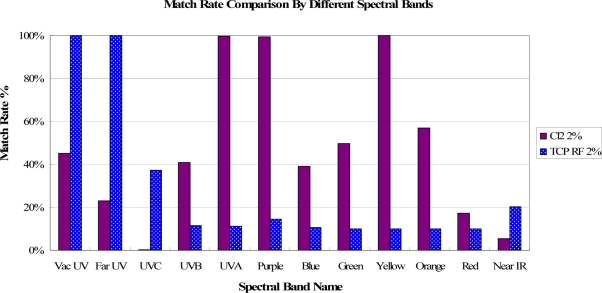
An example of the fault event of Cl_2_ 2% shift versus that of TCP RF power 2% shift.

**Figure 6.**
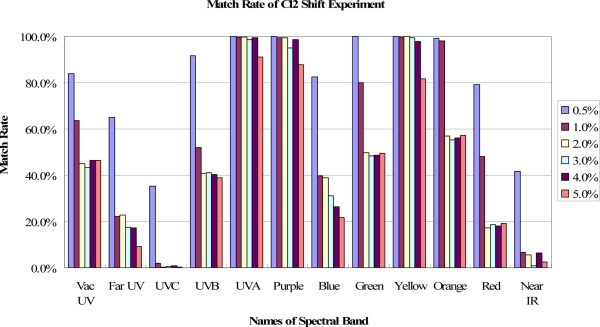
An example for the match rate of Cl_2_ shift experiment. The match rate of each spectral band decreased due to the increasing of the process shift amount.

In Figure 4, the spectral band of OES can be divided into 12 bands, from “Vac UV band with wavelength below 190 nm” to “Near IR band with wavelength above 750 nm”. After deeply analyzing the match rate in each band, it is found that the match rates have different spectral band distributions for different fault events. For example, the fault event of Cl_2_ 2% shift has a different match rate distribution from that of TCP RF power 2% shift, as shown in Figure 5. In addition, these match rates vary depending on the amount of shift for each fault case. The match rates of the spectral bands are nearly decreased by increasing shifts for the same spectral band, while the decreased amounts are different for each spectral band, as shown in Figure 6.

**Figure 7.**
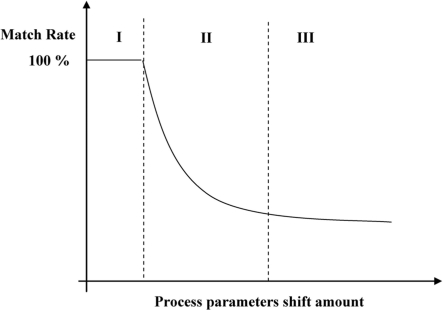
Down trend of matching rate with increasing the shift amount of process parameters.

Figure 7 shows a down trend of the match rates in Ranges II & III when the amount of shift of the process parameters increases. In Range I, the match rate is high and kept constant, meaning that the fault is not detected. As indicated in Range II, the match rate decreases as the process shift becomes larger. The match rate continuously decreases until it is eventually saturated when the shift is larger than a specific amount, as shown in Range III, depending on different process fault. Due to the limited resources, the whole range of fault event data from the conducted experiments cannot be obtained in Ranges I, II, and III. This fault classification model can be performed when the process parameters shift within a known range and the smaller process shift events will be ignored. The proposed method is useful since it can be exploited to identify or/and to classify the causes of fault process and thus to reduce the down time of process tool by decreasing the troubleshooting time.

#### 2.3.1. Fault Classification Modeling

As shown in Ranges II and III of Figure 7, the matching rate of the fault event is decreased or saturated if the process parameter shift amount increases. However, the profile of trends in the spectral bands may be different for every single fault. Some fault events may have unique distributions. Therefore, if a statistical method can be used to identify each trend of the spectral bands, a fault model can be built to classify the fault process type. From the results of the sigma model, the match rate trends, which changed with the increase of the process parameters, can be split into three types: A, B and C. The matching rate in Type A cannot be detected, as shown in Range I. Any curve which covers at least two regions is called Type B. If the data are all located only in Range III, it is called Type C.

Due to different type trends of match rates for each spectral band, it needs two different statistical tools to fit the match rate curves. For Types A and C, the match rate is stable and is within a single range. A general statistical method is thus applied to determine the values of mean ξ and standard deviation σ. For Type B, either the second-order polynomial regression method:

(1) $$y={ax}^{2}+bx+c$$

or exponential regression method:

(2) $$y={hx}^{k}$$

with least-square-error is exploited to fit the curves for the spectral bands. Terms *x* and *y* are known values and represent the parameter shift amount and match rate, respectively. Parameters *a*, *b*, *c*, *h* and *k* are obtained using the regression methods with least-square-error. After these two regression curves, Equations (1) and (2), are fit, the higher R-square value regression curve is selected inside the model table. Table 1 lists six different type of fault events, which include “Cl_2_ gas flow shift +0.5% to 5%”, “Cl_2_ and HBr gas flow shift +0.5% to 5%”, “Cl_2_ and CF_4_ gas flow shift +0.5% to 5%”, “TCP RF power shift +0.5% to 5%”, “ Bottom RF power shift +0.5% to 5%” and “ Chamber pressure shift +2% to 25%”.

#### 2.3.2. Probabilistic Index (PI) Calculation

After building the statistical models, the fault event spectral bands data are sent into the model database for the calculation of the probabilistic index. For Type A and C curves, the probabilistic indexes are defined by ξ and σ. For the testing data, which is equal to ξ, the probabilistic index of band (PIB) value is equal to 1. For the testing data between ξ ± 0.25σ, the PIB value is assigned as 0.9. Similarly, for the testing data between ξ ± 0.5σ, ξ ± σ, ξ ± 2σ, and ξ ± 3σ, the PIB values is given to 0.7, 0.5, 0.3, and 0.1, respectively. Otherwise, the testing data is outside ξ ± 3σ, the PIB is defined to be zero. These PIB numbers are determined, based on the long-term working experience of author. The algorithm of Rule Base 1 for the PIB calculation of Type A or C is expressed as follows:

IF the testing data = ξ THEN PIB = 1

  ELSEIF ξ − 0.25σ ≦ the testing data ≦ ξ + 0.25σ

    THEN PIB = 0.9

  ELSEIF ξ − 0.5σ ≦ the testing data ≦ ξ + 0.5σ

  THEN PIB = 0.7

  ELSEIF ξ − σ ≦ the testing data ≦ ξ + σ

  THEN PIB = 0.5

  ELSEIF ξ − 2σ ≦ the testing data ≦ ξ + 2σ

   THEN PIB = 0.3

  ELSEIF ξ − 3σ ≦ the testing data ≦ ξ + 3σ

   THEN PIB = 0.1

  ELSEIF PIB = 0;

(Rule Base 1)

For the PIB calculation of Type B, the *x* values need to be calculated using the regression methods of Equation (1) or (2) with the known parameters from the fault type model. If the number of spectral bands, which belong to Type B in the fault model, is less than 4 and *x* values are within the fault model forecast range (FR), the PIB value is assigned to be 0.6, otherwise it is zero. For example, in a Cl_2_ gas shift experiment, the FR is from 0 to 0.05. For the number of spectral bands higher than or equal to 4, the mean and standard deviation values of *x* need to be computed due to the greater statistical population. If the mean value is within the forecast range of the fault model, the mean factor (M) is equal to 1, otherwise it is zero. On the other hand, if the standard deviation value of *x* is less than half of the forecast range, SD factor is assigned to 1, otherwise it is zero. The explanation is followed. As the standard deviation value of *x* is larger than half of the forecast range, the regression results indicate that they have different shift amounts of process parameters or are the different fault events in these spectral bands of Type B. After the M and SD values are obtained, the PIB of a spectral band can be obtained utilizing the following algorithm, Rule Base 2,

IF | *x* – M | ≦ (0.05 FR) THEN PIB = M · SD

  ELSEIF | *x* – M | ≦ (0.1FR) THEN PIB = 0.8 M · SD

    ELSEIF | *x* – M | ≦ (0.2FR) THEN PIB = 0.6 M · SD

      ELSEIF | *x* – M | ≦ (0.4FR) THEN PIB = 0.3 M · SD

        ELSEIF | *x* – M | ≦ (1.0FR) THEN PIB = 0.1 M · SD

  ELSEIF PIB = 0;

(Rule Base 2)

**Figure 8.**
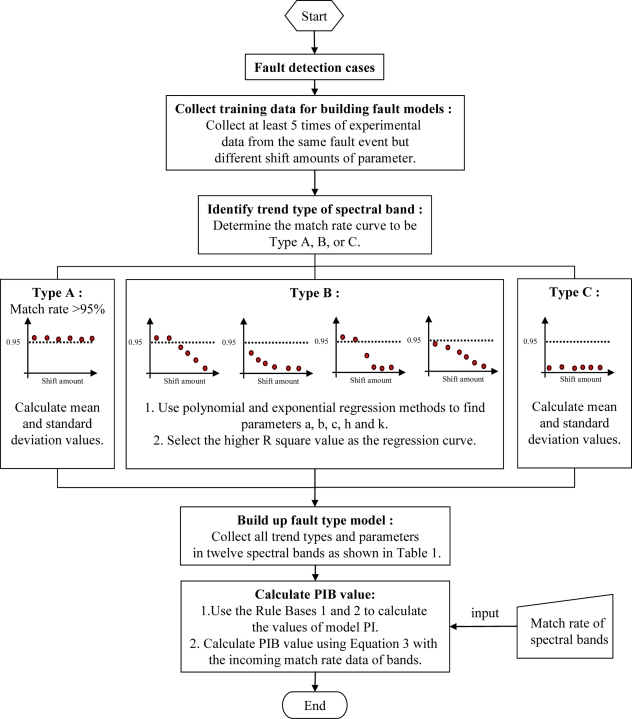
Flowchart of building a fault type model.

Finally, we can calculate the overall PI value of fault type model from the following equation:

(3) $$\mathrm{PI}=\frac{\sum_{\mathrm{Band}=\mathrm{VacUV}}^{\mathrm{NearIR}}\mathrm{PI}B_{\mathrm{Band}}\times \mathrm{Band}\;\mathrm{Range}\;\mathrm{Pixe}l_{\mathrm{Band}}}{2048\;\mathrm{Pixel}}$$

where Band Range Pixel represents the assigned spectral band, sharing how many pixels of the CCD sensors, and the sub-index of Band Range Pixel “Band” indicates which of the spectral bands, shown in Figure 4, is calculated. Figure 8 shows the flowchart of building a fault type model. First, we collect at least five sets of experimental data, which are at the same event but with the different process shift amounts. Then, we use these data to determine the band trend type; that is, Type A, B, or C. Next, we utilize the statistical regression methods to find the fault type model. Finally, Rule Bases 1 and 2 are applied to calculate the probabilistic index of the fault type event.

#### 2.3.3. Event Type Classification by Using Probabilistic Index

**Figure 9.**
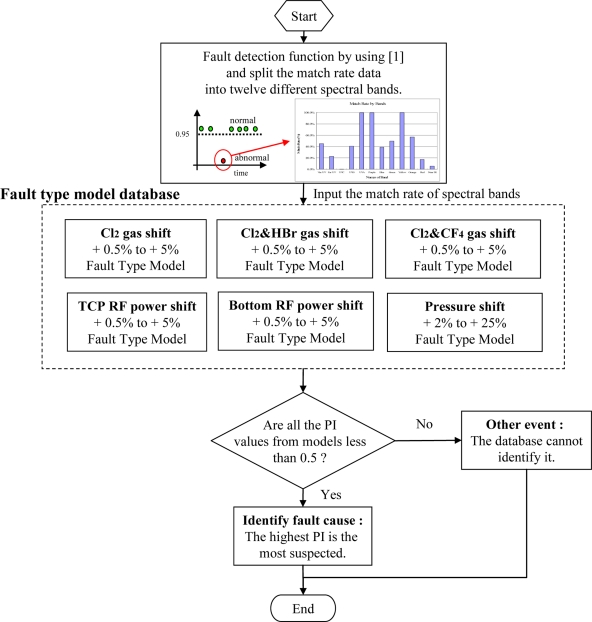
Flowchart of event classification using the probabilistic index for each fault type model.

After building up several different models of fault type off-line using the proposed method, the fault events can be classified as described in the flowchart in Figure 9. The fault plasma process is automatically detected by the sigma match rate method in real-time [12]. The data of match rate are split into 12 bands with different spectral bands. Then, each fault type model receives the 12 split match rate data to calculate its PI value. Finally, among the six calculated PI values from the six built models, the highest PI value is regarded as the most probable root cause of the fault. Once the possible clue is found, the downtime is reduced and the productivity is promoted.

## 3. Results and Discussion

**Figure 10.**
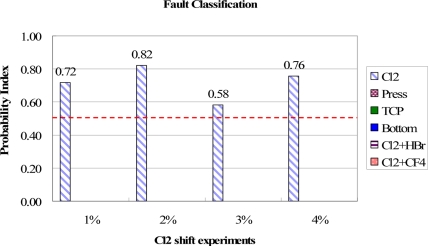
Test results of fault classification for Cl_2_ gas flow shift. The Cl_2_ fault events are clearly identified from highest PI values.

In this work, six different types of fault model were built by the sigma match rate method in real-time [12]. The experiments were conducted to obtain the match rate data, used to verify the ability of fault type classification by the proposed method, except for the non-detected cases HBr and CF_4_ gas flow shifts. Based on the match data, the PI values are calculated, from which the highest PI is marked to be the most possible fault type. Figure 10 shows the testing results of Cl_2_ gas flow shift, from 1% to 4%, in which the highest PI value of each one-percent shift is presented. In the figure, the PI values of other fault models are all zero because the Cl_2_ fault model belongs to Type B and has the totally different trend type curves from other models as indicated in Table 1. Thus the Cl_2_ fault model is correctly classified. This unique combination of trend type curves also leads the Cl_2_ fault model have very low PI values in performing the experiments of other type fault events.

**Figure 11.**
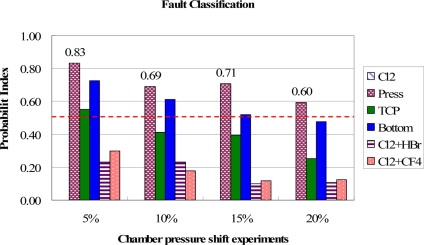
Test results of fault classification for chamber pressure shift experiments. The chamber pressure fault events are clearly identified from highest PI values.

The test results for the chamber pressure fault events are shown in Figure 11. The highest PI values are presented to correctly identify that the most possible fault is caused by chamber pressure, though all the PI values of bottom RF power shift are close to those of chamber pressure in the event tests. It is observed that the types of trend curves for each spectral band of these two fault events are the same as shown in Table 1, but the means and standard deviation are slightly different from some spectral bands. On the other hand, the gas flow shift events, including Cl_2_ and HBr and Cl_2_ and CF_4_, have relatively lower PI values due to their trend curve types of match rate are much different from the chamber pressure shift. Again the Cl_2_ fault type model shows zero PI values in the tests.

**Figure 12.**
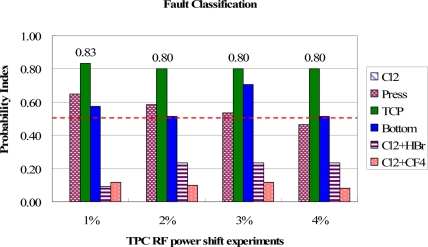
Test results of fault classification for TCP RF power shift experiments. The TCP RF power fault events are clearly identified from highest PI values.

In Figure 12, the TCP RF power shift events are all clearly classified from other fault events. The PI values of TCP RF power shifts are all larger than 0.8, indicating that the proposed method can confidently classify the TCP RF power shift among other fault events. Again, the gas flow shift groups show lower PI values than other groups.

**Figure 13.**
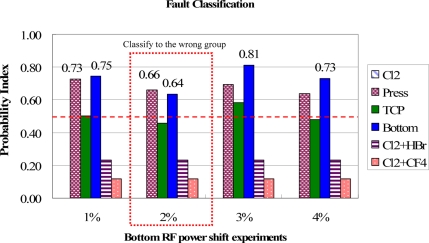
Test results of fault classification for the bottom RF power shift experiments. The bottom RF power fault events are clearly identified from highest PI values.

**Figure 14.**
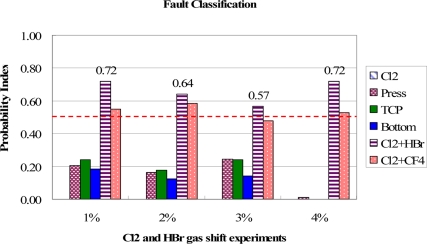
Test results of fault classification for the Cl_2_ & HBr gas flow shift experiments. The Cl_2_ and HBr gas flow fault events are clearly identified from highest PI values.

**Figure 15.**
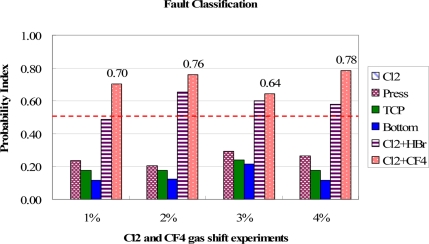
Test results of fault classification for the Cl_2_ & CF_4_ gas flow shift experiments. The Cl_2_ and CF_4_ gas flow fault events are clearly identified from highest PI values.

In Figure 13, the classification results for the bottom RF power shift are shown. Except for the 2% shift, the bottom RF power cause can be distinguished from other groups. However, in the 2% shift, the most possible fault is wrongly assigned to a chamber pressure shift due to the same combination of trend curve types in these two groups. This may result in a wrong classification if the fault events have a similar combination of fault models. It therefore suggested that if the difference between the highest and second highest PI values is lower than 0.05, the second fault event should be also considered as the major cause for the troubleshooting. Further investigation has to be carried out to solve this problem. But, at least, it cuts the number of potential causes down to two, even though these two groups have the same combination of trend curve types. As a result, the proposed method still can reduce the time needed to find the root causes and saves downtime recovery costs. The test results of gas flow shift events for Cl_2_ and HBr and Cl_2_ and CF_4_ are shown in Figures 14 and 15, respectively. The proposed method can classify these fault events from the highest PI values.

**Figure 16.**
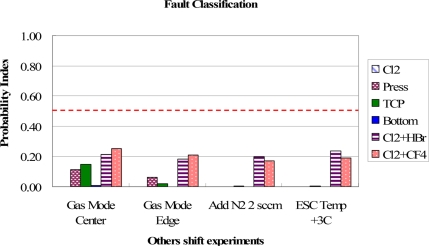
Test results of fault classification for the excluded event shift experiments. The proposed method can be classified them into “other” fault cases due to all highest PI values are lower than 0.5.

To validate the proposed method, some fault events, which were not constructed in the database of fault models, were employed to test the classification function. The test results for Gas Mode Center [12], Gas Mode Edge [12], adding N_2_ into chamber, and increased ESC temperature are shown in Figure 16, where all the highest PI values for the excluded fault events are much lower than 0.5, meaning that these test fault events are successfully classified as unknown fault events. To test the fault classification of adding N_2_ gas, it shows zero PI values for the shift events of Cl_2_ gas, chamber pressure, TCP RF power, and bottom RF power. Similar results are seen in the test event of increasing the ESC temperature. It points out that these testing events have totally different match rate distributions from the fault type models mentioned. From the experimental results shown in Figures 10–16, we can conclude that the overall accuracy rate of the classification for fault event shifts is 27 out of 28 or about 96.4% success. Moreover, the proposed method can classify the fault causes of plasma etching in real-time and easily build up the fault models.

## 4. Conclusions

The plasma process tool is often used in the semiconductor fabrication etching process. In order to maximize the product/wafer yield and tool productivity, a timely and effective fault process detection and classification method is required in a plasma reactor. The classification of fault events can help end users to quickly identify the fault process, and thus to reduce the plasma tool downtime. In this work, a real-time classification method of fault cause is developed. Based on the match rates obtained from our previous study [12], six fault models are constructed in a database by using simple statistical method. Probabilistic index (PI) is defined to identify the most probable root cause for a fault event. Experiments were conducted to test the fault classification results. From the experimental results, it can be concluded that the proposed fault classification method is feasible and the overall accuracy rate of the classification for fault event shifts is 27 out of 28 or about 96.4% success. Moreover, the fault models can be easily built to classify the plasma etching fault causes in real-time.

## Figures and Tables

**Table 1.** Six different types of fault events with the values of its model parameters.

(a) Fault Event of Cl_2_ Model with Gas Shift 0.5% to 5%.								
	**Trend Type**	**ξ**	**σ**	**a**	**b**	**c**	**h**	**k**
VAC UV	B	NA	NA	435.45	−30.583	0.9364	NA	NA
FAR UV	B	NA	NA	NA	NA	NA	0.0159	−0.6618
UVC	B	NA	NA	340.22	−23.35	0.3556	NA	NA
UVB	B	NA	NA	NA	NA	NA	0.1262	−0.3417
UVA	B	NA	NA	−82.903	3.0422	0.9786	NA	NA
Purple	B	NA	NA	−78.194	2.0874	0.9847	NA	NA
Blue	B	NA	NA	NA	NA	NA	0.0507	−0.5021
Green	B	NA	NA	549.54	−39.993	1.1536	NA	NA
Yellow	B	NA	NA	−192.66	7.3656	0.9516	NA	NA
Orange	B	NA	NA	500.33	−37.255	1.2064	NA	NA
Red	B	NA	NA	654.84	−46.728	0.938	NA	NA
Near IR	B	NA	NA	NA	NA	NA	0.0007	−1.1123

(b) Fault Event of Cl2 + HBr Model with Gas Shift 0.5% to 5%.								
	**Trend Type**	**ξ**	**σ**	**a**	**b**	**c**	**h**	**k**
VAC UV	A	0.999	0.003	NA	NA	NA	NA	NA
FAR UV	A	0.987	0.018	NA	NA	NA	NA	NA
UVC	B	NA	NA	−208.6	7.781	0.357	NA	NA
UVB	C	0.162	0.004	NA	NA	NA	NA	NA
UVA	C	0.162	0.010	NA	NA	NA	NA	NA
Purple	C	0.186	0.009	NA	NA	NA	NA	NA
Blue	C	0.146	0.004	NA	NA	NA	NA	NA
Green	C	0.140	0.000	NA	NA	NA	NA	NA
Yellow	C	0.140	0.000	NA	NA	NA	NA	NA
Orange	C	0.140	0.000	NA	NA	NA	NA	NA
Red	C	0.140	0.000	NA	NA	NA	NA	NA
Near IR	B	NA	NA	−30.464	−0.0936	0.213	NA	NA

(c) Fault Event of Cl_2_+CF_4_ Model with Gas Shift 0.5% to 5%.								
	**Trend Type**	**ξ**	**σ**	**a**	**b**	**c**	**h**	**k**
VAC UV	A	1.000	0.000	NA	NA	NA	NA	NA
FAR UV	A	1.000	0.000	NA	NA	NA	NA	NA
UVC	B	NA	NA	−6.552	−3.778	0.504	NA	NA
UVB	C	0.161	0.004	NA	NA	NA	NA	NA
UVA	C	0.166	0.011	NA	NA	NA	NA	NA
Purple	C	0.191	0.009	NA	NA	NA	NA	NA
Blue	C	0.147	0.003	NA	NA	NA	NA	NA
Green	C	0.140	0.000	NA	NA	NA	NA	NA
Yellow	C	0.140	0.000	NA	NA	NA	NA	NA
Orange	C	0.140	0.000	NA	NA	NA	NA	NA
Red	C	0.140	0.000	NA	NA	NA	NA	NA
Near IR	C	0.211	0.003	NA	NA	NA	NA	NA

(d) Fault Event of TCP RF Power Model with Power Shift 0.5% to 5%.								
	**Trend Type**	**ξ**	**σ**	**a**	**b**	**c**	**h**	**k**
VAC UV	A	1.000	0.000	NA	NA	NA	NA	NA
FAR UV	A	0.9995	0.001	NA	NA	NA	NA	NA
UVC	B	NA	NA	11.943	−1.044	0.389	NA	NA
UVB	C	0.116	0.001	NA	NA	NA	NA	NA
UVA	C	0.114	0.001	NA	NA	NA	NA	NA
Purple	C	0.143	0.001	NA	NA	NA	NA	NA
Blue	C	0.105	0.000	NA	NA	NA	NA	NA
Green	C	0.100	0.000	NA	NA	NA	NA	NA
Yellow	C	0.100	0.000	NA	NA	NA	NA	NA
Orange	C	0.100	0.000	NA	NA	NA	NA	NA
Red	C	0.101	0.000	NA	NA	NA	NA	NA
Near IR	B	NA	NA	19.745	−2.5749	0.2452	NA	NA

(e) Fault Event of Bottom RF Power Model with Power Shift 0.5% to 5%.								
	**Trend Type**	**ξ**	**σ**	**a**	**b**	**c**	**h**	**k**
VAC UV	A	1.000	0.0000	NA	NA	NA	NA	NA
FAR UV	A	1.000	0.0000	NA	NA	NA	NA	NA
UVC	C	0.403	0.0147	NA	NA	NA	NA	NA
UVB	C	0.116	0.0019	NA	NA	NA	NA	NA
UVA	C	0.115	0.0043	NA	NA	NA	NA	NA
Purple	C	0.143	0.0026	NA	NA	NA	NA	NA
Blue	C	0.105	0.0023	NA	NA	NA	NA	NA
Green	C	0.100	0.0000	NA	NA	NA	NA	NA
Yellow	C	0.100	0.0000	NA	NA	NA	NA	NA
Orange	C	0.100	0.0000	NA	NA	NA	NA	NA
Red	C	0.100	0.0000	NA	NA	NA	NA	NA
Near IR	C	0.189	0.0114	NA	NA	NA	NA	NA

(f) Fault Event of Chamber Pressure Model with Pressure Shift 2% to 25%.								
	**Trend Type**	**ξ**	**σ**	**a**	**b**	**c**	**h**	**k**
VAC UV	A	1.000	0.0000	NA	NA	NA	NA	NA
FAR UV	A	1.000	0.0000	NA	NA	NA	NA	NA
UVC	C	0.380	0.0267	NA	NA	NA	NA	NA
UVB	C	0.120	0.0046	NA	NA	NA	NA	NA
UVA	C	0.121	0.0115	NA	NA	NA	NA	NA
Purple	C	0.147	0.0084	NA	NA	NA	NA	NA
Blue	C	0.105	0.0037	NA	NA	NA	NA	NA
Green	C	0.100	0.0000	NA	NA	NA	NA	NA
Yellow	C	0.100	0.0000	NA	NA	NA	NA	NA
Orange	C	0.100	0.0000	NA	NA	NA	NA	NA
Red	C	0.100	0.0000	NA	NA	NA	NA	NA
Near IR	C	0.214	0.0119	NA	NA	NA	NA	NA
